# Molecular Detection of Yellow Fever Virus in *Haemagogus janthinomys* Mosquitoes (Diptera: Culicidae) in a Rural Settlement in the State of Pará, Brazilian Amazon, 2024

**DOI:** 10.3390/v17091258

**Published:** 2025-09-18

**Authors:** Joaquim Pinto Nunes Neto, Daniel Damous Dias, Bruna Laís Sena do Nascimento, Sandro Patroca da Silva, Sâmia Luzia Sena da Silva, Lúcia Aline Moura Reis, Hanna Carolina Farias Reis, Fábio Silva da Silva, Lucas Henrique da Silva e Silva, Durval Bertram Rodrigues Vieira, Roberto Carlos Feitosa Brandão, Wallace Oliveira Rosário, Francisco Amilton dos Santos Paiva, José Wilson Rosa Júnior, Bruno Tardelli Diniz Nunes, Lívia Carício Martins, Lívia Medeiros Neves Casseb, Ana Cecília Ribeiro Cruz

**Affiliations:** 1 Department of Arbovirology and Hemorrhagic Fevers, Evandro Chagas Institute, Secretariat of Health and Environment Surveillance, Ministry of Health, Ananindeua 67030-000, PA, Brazil; danieldias@iec.gov.br (D.D.D.); brunanascimento@iec.gov.br (B.L.S.d.N.); sandrosilva@iec.gov.br (S.P.d.S.); samialuzia23@gmail.com (S.L.S.d.S.); h.carolinafreis@gmail.com (H.C.F.R.); fabiodasilva@iec.gov.br (F.S.d.S.); lucashenriqueuepa@gmail.com (L.H.d.S.e.S.); durvalvieira@iec.gov.br (D.B.R.V.); robertobrandao@iec.gov.br (R.C.F.B.); wallacerosario@iec.gov.br (W.O.R.); franciscopaiva@iec.gov.br (F.A.d.S.P.); josejr@iec.gov.br (J.W.R.J.); brunonunes@iec.gov.br (B.T.D.N.); liviamartins@iec.gov.br (L.C.M.); liviacasseb@iec.gov.br (L.M.N.C.); anacecilia@iec.gov.br (A.C.R.C.); 2 Graduate Program in Parasitary Biology in the Amazon, Center of Biological and Health Sciences, State University of Pará, Belém 66095-663, PA, Brazil; 3 Faculty of Nursing, Federal and Rural University of Amazon, Parauapebas 68515-000, PA, Brazil; lucia.reis@ufra.edu.br

**Keywords:** arbovirus, entomovirological investigation, RT-qPCR, metagenomics

## Abstract

Yellow fever (YF) is an acute and potentially fatal hemorrhagic disease caused by the Yellow Fever virus (YFV), endemic to sub-Saharan Africa and several tropical countries, including Brazil. In Brazil, the Amazon region is considered the main endemic area. YFV is maintained in a sylvatic cycle involving Neotropical primates and mosquitoes of the genera *Haemagogus* and *Sabethes*, acting as primary and secondary vectors, respectively. In March 2024, entomovirological surveillance was conducted in Santa Bárbara do Pará, Pará, Brazil. A total of 286 mosquitoes were collected, classified into 13 species across nine genera, and grouped into 33 pools. Seventeen pools were tested by RT-qPCR for *Orthoflavivirus* (YFV, DENV, WNV, SLEV), *Alphavirus* (CHIKV, MAYV), and *Orthobunyavirus* (OROV). YFV was detected in four *Haemagogus janthinomys* pools, with Ct values ranging from 22.2 to 27.9. Metagenomic sequencing confirmed the presence of YFV with assigned reads and >99% protein identity. Notably, the detection occurred without human cases or primate deaths, enabling timely vaccination of the local population. These findings confirm YFV circulation in forested areas of the Belém metropolitan region and reaffirm *Hg. janthinomys* as a key vector. Our study reinforces the relevance of early entomovirological surveillance and preventive strategies, such as vaccination, to mitigate yellow fever reemergence.

## 1. Introduction

Yellow fever (YF) is a viral vector-borne disease that causes an acute and potentially fatal hemorrhagic fever, with significant impact on public health [1,2]. It occurs in sub-Saharan Africa and in several tropical and subtropical countries in South America, including Brazil. Despite the availability of a safe and effective vaccine, it still remains a major public health concern in these regions, where epidemic outbreaks occur intermittently [3,4].

YF is caused by *Orthoflavivirus flavi*, an enveloped virus with a positive-sense, single-stranded, non-segmented RNA genome, classified into the genus *Orthoflavivirus*, family *Flaviviridae* [1]. In the Americas, YF transmission occurs through two distinct epidemiological cycles: sylvatic and urban. In both cycles, the disease is identical in clinical, immunological, and pathophysiological aspects [5]. In the sylvatic cycle, non-human primates, particularly those of the genera *Allouata* Lacépède, *Ateles* Linnaeus, *Callithrix* Linnaeus, and *Cebus* Erxleben [6,7,8], act as the main amplifying hosts of the virus, while mosquito species of the genera *Haemagogus* Williston and *Sabethes* Robineau-Desvoidy serve as primary and secondary vectors, respectively [9,10,11]. Human infection is accidental and occurs when unvaccinated individuals living in or entering forested areas for extractive or recreational activities are bitten by infected mosquitoes. In the urban cycle, viremic humans infected in forested areas may return to urban environments and serve as a source of infection for anthropophilic mosquitoes such as *Aedes aegypti*. Although urban cases have not been reported in the Americas since 1954, they remain common in several African countries [4].

In Brazil, the introduction of the 17D vaccine in the late 1930s, combined with mosquito eradication campaigns targeting *Aedes aegypti*, led to a dramatic decline in urban YF cases and the eventual elimination of this transmission cycle [5,12,13]. The last recorded urban cases occurred in 1942, in the municipality of Sena Madureira, in the state of Acre. Despite this, sylvatic YF continued to occur in endemic areas of the Amazon region. However, starting in 1999, a concerning shift in the distribution pattern of the disease was observed. For the first time, the majority of human cases and non-human primates were reported outside the Amazon basin, affecting states in the Central-West, Southeast, and South of the country [5,14,15,16]. These outbreaks occurred in areas near regions with high infestation levels of the urban vector *Aedes aegypti* and the potential vector *Aedes albopictus*, close to large, densely populated metropolitan centers. These areas were also characterized by low vaccination coverage [17].

In recent decades, the dynamics of YF in Brazil have raised concerns among public health authorities, prompting discussions about the risk of reestablishment of urban transmission in the country [18,19,20,21]. In this context, the individuals most susceptible to contracting the disease are those who are unvaccinated and exposed to infected vectors in endemic areas with active viral circulation, particularly in forested and rural environments [9]. It is important to highlight that, although the Amazon region is characterized by irregular and sporadic patterns of YF cases usually associated with self-limited outbreaks and epidemics [21] it holds great epidemiological importance as the natural reservoir of the disease in Brazil. Historically, the region has been the origin of outbreak waves that spread to other parts of the country, including the South and Southeast [22,23].

In this context, entomovirological surveillance stands out as an essential tool for the early detection of viral circulation and the identification of areas with transmission potential, based on the occurrence of epizootics in non-human primates, suspected human cases, and the identification of potential vector species. Such actions are fundamental for predicting and stratifying the risk of yellow fever emergence, thereby supporting the formulation of effective control and prevention measures [24,25,26].

Considering the relevance of the Amazon region as one of the largest arbovirus reservoirs on the planet [27], and particularly as the primary endemic reservoir of YFV in Brazil [23], this scenario highlights the region’s vulnerability to arboviral emergencies. The study area, for instance, has a concerning history of arbovirus outbreaks. In 2008, an outbreak of *Alphavirus mayaro* (MAYV) was recorded with 36 confirmed cases, with *Hg. janthinomys* identified as the main vector involved in transmission [28]. A decade later, in 2018, another episode affected 94 individuals, with a predominance of *Orthobunyavirus oropoucheense* (OROV) infections, although cases of MAYV and *Alphavirus chikungunya* (CHIKV) were also reported [29]. In this context, it is imperative to carry out and disseminate official records of viral detections, as well as the identification of the mosquito species involved. The data presented in this study contribute to a better understanding of the geographical distribution of YFV in the Amazon region and to the improvement of prevention strategies, including the expansion of vaccination coverage.

This study reports the molecular detection of yellow fever virus (*Orthoflavivirus flavi*) in pools of *Haemagogus janthinomys* mosquitoes collected from a rural area in the metropolitan region of Belém, the capital of the state of Pará, in the Brazilian Amazon, during entomovirological investigations conducted in March 2024.

## 2. Materials and Methods

### 2.1. Study Area

**Figure 1:**
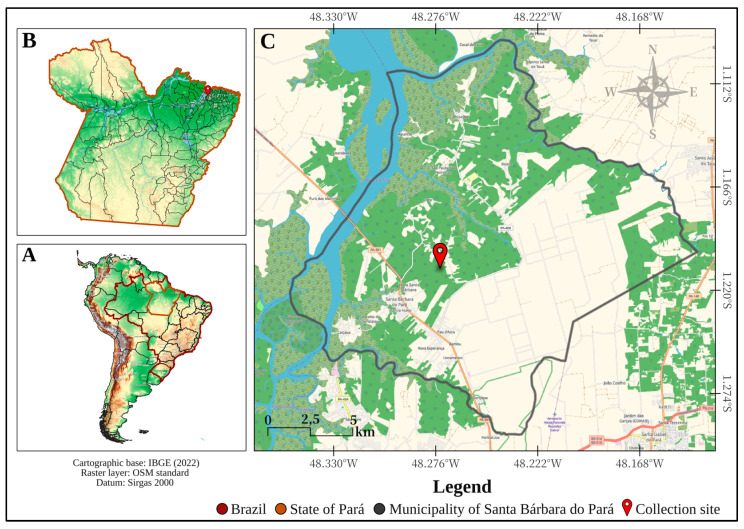
Location map of the municipality of Santa Bárbara do Pará, highlighting the collection site of the investigated mosquito species. The figure was created using QGIS v.3.22 (https://qgis.org/en/site/, accessed on 6 July 2025) with cartographic data from the Brazilian Institute of Geography and Statistics (IBGE, 2022) (https://www.ibge.gov.br/, accessed on 6 July 2025) and raster layers from the OpenStreetMap project (https://www.openstreetmap.org/, accessed on 6 July 2025) Panel (**A**) shows the state of Pará; panel (**B**) shows South America, with Brazil outlined in red and Pará outlined in orange; and panel (**C**) shows the boundaries of the municipality of Santa Bárbara do Pará, indicating the collection site with a red marker.

The study was conducted at the “Expedito Ribeiro” Agroecological Rural Workers Settlement, located at coordinates 1°12′29.73″ S and 48°16′25.25″ W (Figure 1), in the municipality of Santa Bárbara do Pará, State of Pará, Brazilian Amazon, between 19 and 21 March 2024. The research was carried out under authorization granted by the Biodiversity Authorization and Information System of the Ministry of the Environment (SISBIO/IBAMA), permit no. 56504-6. The settlement covers approximately 600 hectares, of which 80% are designated as legal forest reserve. Local residents rely economically on agricultural production, small-scale livestock farming, and beekeeping.

The region’s climate is classified as hot and humid tropical, with significant rainfall throughout most of the year. According to the Köppen–Geiger classification, it falls into the Am category. The annual average temperature is 26.4 °C, with a relative humidity of 85%, and total annual precipitation reaches approximately 2.624 mm. The rainy season extends from December to May, while lower precipitation levels are observed between July and November (https://en.climate-data.org/, accessed on 8 June 2025).

### 2.2. Mosquito Sampling and Taxonomic Identification

Sampling was conducted simultaneously in two forest strata: at ground level and in the forest canopy, approximately 12 m high. A single collector was positioned in the canopy, while two others operated at ground level. Mosquitoes were collected using the protected human attraction technique [30], with the aid of small entomological nets and oral aspirators. Specimens were then transferred to cryogenic tubes and immediately frozen in liquid nitrogen at −196 °C, and transported to the Department of Arbovirology and Hemorrhagic Fevers of the Evandro Chagas Institute (SEARB/IEC/SVSA/MS), where they were stored at −70 °C until taxonomic identification.

At the Medical Entomology Laboratory (SEARB/IEC), specimens were identified based on external morphology using the dichotomous key proposed by Forattini [31], with the aid of a Zeiss Stemi 2000-C stereomicroscope (Carl Zeiss, Göttingen, Germany) on a cold table (Eletrohospitalar, Brasília, Federal District, Brazil) maintained at approximately −38 °C. Genus and subgenus abbreviations followed the conventions proposed by Reinert [32]. After identification, specimens were grouped into pools containing 1 up to 33 individuals, according to species, genera (female and male), collection site, strata and date. Each pool received a unique alphanumeric code beginning with “AR” (for *ARthropod* samples), followed by sequential numbers.

### 2.3. Mosquito Homogenization and Viral RNA Extraction

Pools containing the entire bodies of the insects were homogenized in 1 mL of Dulbecco’s Phosphate-Buffered Saline (DPBS) (Gibco, Waltham, MA, USA) supplemented with 5% fetal bovine serum, 2% penicillin-streptomycin (Sigma-Aldrich, Burlington, MA, USA), and 1% amphotericin B (Sigma-Aldrich, Burlington, MA, USA), along with a 5 mm tungsten bead (Qiagen, Hilden, Germany). The pools were then macerated by agitation in a Tissuelyser II (Qiagen, Hilden, Germany) at a frequency of 25 Hz for 1 min and stored at −70 °C for 24 h. Subsequently, the samples were thawed and centrifuged at 10,000 rpm for 10 min at 4 °C, and 140 μL of the supernatant was collected for total RNA extraction and purification using the QIAamp Viral RNA^®^ Kit (Qiagen, Hilden, Germany), following the manufacturer’s instructions.

### 2.4. Real-Time Reverse Transcription Polymerase Chain Reaction (RT-qPCR) Assay

The one-step real-time reverse transcription polymerase chain reaction (RT-qPCR) assay was performed using the SuperScript^®^ III Platinum^®^ One-Step qRT-PCR Kit (ThermoFisher Scientific, Waltham, MA, USA). The test for *Orthoflavivirus denguei* (DENV) was carried out as a single-tube multiplex assay capable of detecting all four virus serotypes. Assays for YFV, *Orthoflavivirus nilense* (WNV), *Orthoflavivirus louisense* (SLEV), *Alphavirus chikungunya* (CHIKV), *Alphavirus mayaro* (MAYV), and *Oropouche orthobunyavirus* (OROV) were conducted in duplicate as singleplex reactions. Each run included positive and negative controls, as well as a Non-Template Control (NTC), to validate the reactions. Amplifications were carried out on an ABI 7500 Fast Real-Time PCR System (Applied Biosystems, Thermo Fisher Scientific, Waltham, MA, USA). Thermal cycling conditions for the RT-qPCR assays were as follows: reverse transcription at 50 °C for 30 min; initial denaturation at 95 °C for 2 min; followed by 45 cycles of 15 s at 95 °C and 1 min at 60 °C.

Virus detection was based on specific primers and probes according to previously established protocols: YFV as described by Fischer et al. [33]; DENV according to Santiago et al. [34]; SLEV per Lanciotti and Kerst [35]; WNV following Lanciotti et al. [36]; CHIKV as described by Lanciotti [37]; and MAYV and OROV according to Naveca et al. [38].

Samples were considered positive when the average cycle threshold (Ct) value was ≤37 for YFV and DENV, and ≤38 for WNV, SLEV, CHIKV, MAYV, and OROV. None of the positive pools in this study exhibited late amplification. The RT-qPCR protocol employed was validated by the Brazilian National Reference Laboratory for Arbovirus Diagnostics (SEARB/IEC) and has a reported limit of detection ranging from 4.0 to 8.8 RNA copies per reaction [33]. The Ct cut-off was determined using quantified samples at the Limit of Detection (LoD) concentration, tested in 95 replicate reactions. It was defined as the mean Ct plus one standard deviation (≈38). Samples with Ct values >38 were considered negative, while those between 35 and 38 were re-tested using a doubled input volume; a reduction of ≥1 Ct confirmed positivity.

Viral RNA loads in the positive samples were estimated using a standard curve generated from a YFV-positive sample previously quantified by RT-droplet digital PCR (RT-ddPCR), ranging from 6.22 × 10^2^ to 2.67 × 10^4^ RNA copies per reaction.

### 2.5. Metagenomic Sequencing

#### 2.5.1. Library Preparation and Sequencing

Only virus-positive samples confirmed by RT-qPCR were selected for sequencing. Extracted RNA from these samples was used for first- and second-strand cDNA synthesis using the SuperScript™ VILO™ MasterMix Kit (Thermo Fisher Scientific, Waltham, MA, USA) and the NEBNext mRNA Second Strand Synthesis Module (New England Biolabs, Ipswich, MA, USA). Subsequently, cDNA libraries were constructed following the protocol of the SureSelectQXT Whole Genome Library Prep Kit (Agilent Technologies, Santa Clara, CA, USA). Library quantification was performed using a Qubit^®^ 4.0 fluorometer (Life Technologies, Waltham, MA, USA), while quality assessment was carried out with an Agilent 2100 Bioanalyzer (Agilent Technologies, Santa Clara, CA, USA). The sequencing step was performed on the NextSeq 550 platform (Illumina, San Diego, CA, USA) in paired-end mode using the NextSeq 500/550 High Output Kit v2.5 (300 cycles).

#### 2.5.2. Bioinformatics Analysis

Raw sequence data were subjected to quality control using Fastp v.0.23.0 [39], configured to remove adapter sequences and reads with a Phred quality score below Q20. After this step, the reads were submitted to De novo genome assembly using MEGAHIT v.1.2.9 [40] with multiple k-mer sizes (21, 31, 41, 51, 61, 71, 81, 91, 99, and 141). Taxonomic classification was performed at two levels: (i) on the processed reads and (ii) on the assembled contigs, using Kraken 2 v.2.1.2 [41] with the Core_nt database (https://benlangmead.github.io/aws-indexes/k2, accessed on 7 June 2025). The results were inspected using the online Pavian Metagenomics Data Explorer (https://fbreitwieser.shinyapps.io/pavian/, accessed on 7 June 2025) where identification and quantification of viral reads were carried out. Subsequently, assembled contigs were grouped and mapped to the YFV reference genome (GenBank accession: NC_002031) using Bowtie2 [42]. The resulting alignment files were processed with Samtools v.1.21 [43] to convert file formats and generate a consensus sequence. Finally, the consensus sequence was submitted to BLASTX (https://blast.ncbi.nlm.nih.gov/Blast.cgi, accessed on 7 May 2025) for sequence similarity analysis against the non-redundant (nr) database.

## 3. Results

A total of 286 mosquito specimens belonging to 13 species across nine genera were collected. The majority of specimens were recorded at the ground level, totaling 228 individuals (79.7%), while the canopy accounted for 58 individuals (20.3%) (Table 1).

The most abundant species were *Haemagogus* (*Haemagogus*) *janthinomys* with 77 individuals (26.9%), *Limatus flavisetosus* (*n* = 67; 23.4%), *Wyeomyia* sp. (*n* = 54; 18.9%), *Aedes* (*Ochlerotatus*) *serratus* (*n* = 26; 9.1%), and *Psorophora* (*Janthinosoma*) *ferox* (*n* = 18; 6.3%). Together, these species represented 84.6% of the total mosquitoes collected. The most frequently collected species, *Hg. janthinomys* was collected in both forest strata, being more abundant in the canopy (*n* = 47) than at ground level (*n* = 30). The ground-level fauna exhibited greater species richness and abundance, including 12 taxa found exclusively in this stratum, such as *Ae.* (*Howardina*) *fulvithorax*, *Ae.* (*Och.*) *scapularis*, *Ae.* (*Protomacleaya*) *argyrothorax*, *Li. durhamii*, and *Trichoprosopon* sp. In contrast, only three taxa were recorded exclusively in the canopy: *Haemagogus* (*Conopostegus*) *leucocelaenus* (*n* = 1), *Sabethes* (*Sabethoides*) *chloropterus* (*n* = 7), and *Sa.* (*Sbo.*) *glaucodaemon* (*n* = 2).

Of the 33 pools formed, 17 were selected for RT-qPCR screening based on their composition of species with recognized medical importance and well-established roles in the transmission of the arboviruses under investigation, as well as the availability of standardized protocols for testing arthropod samples (Table 2).

Among the pools tested, yellow fever virus (YFV) was the only virus detected, found in all four *Hg. janthinomys* pools, with cycle threshold (Ct) values ranging from 22.2 to 27.9. The lowest Ct value (22.2), indicating the highest viral load, was observed in a pool composed of 27 individuals collected from the canopy (Supplementary Material S1).

Three *Hg. janthinomys* pools that tested positive for YFV in the RT-qPCR assay were subjected to metagenomic sequencing. A total of 110,939,520 paired-end reads were obtained, of which 107,580,006 were retained after quality control (Table 3). Sample AR888786 was not included in the sequencing process because technical issues occurred during library preparation, which prevented the generation of data with sufficient quality for downstream analyses.

The taxonomic analysis of the reads revealed variation in viral sequence abundance among the analyzed samples (Table 3). Sample AR888776 yielded 50,390 viral reads, of which 54 were assigned to YFV. Samples AR888787 and AR888788 showed 37,007 (12 YFV) and 38,178 reads (3 YFV), respectively. In AR888787, seven contigs ranging from 308 to 560 nucleotides in length were partially assembled and aligned in scattered regions of the YFV reference genome (GenBank accession: NC_002031). In AR888776, five longer partial contigs (368–1149 nt) were obtained; however, genome coverage remained fragmented. In contrast, the limited number of YFV-specific reads in sample AR888788 hindered contig assembly. The partial YFV sequences generated in this study are available as Supplementary Materials S2 and S3.

The assembled contigs showed significant similarity to YFV reference sequences available in GenBank, including strains UVG67813 and AWB15005, both previously reported in Brazil (Table 3). BLASTX analyses indicated partial alignments with the YFV polyprotein, covering 21% to 39% of the reference sequences, with sequence identities of 99.74% and 99.46% and highly significant e-values (0.0 and 5 × 10^−105^), respectively.

## 4. Discussion

Brazil is recognized as an endemic region for YFV, with the Amazonian area serving as a key hotspot due to its diverse natural reservoirs, such as non-human primates and various mosquito vector species [5,23]. The region’s unique ecological and climatic conditions are highly conducive to the persistence of the sylvatic transmission cycle. Its rich biodiversity and complex forest structure facilitate the maintenance and periodic reemergence of YFV in zones previously considered low-risk [19,21,44]. Here, the present study reports the molecular detection of YFV in four pools of *Hg. janthinomys* mosquitoes, collected during entomovirological surveys carried out in March 2024 in a rural settlement within the metropolitan region of Belém, Pará.

The faunal analysis revealed mosquito diversity and distribution, with a predominance of specimens collected at ground level (79.7%) compared to the canopy (20.3%). *Hg. janthinomys* was the most abundant species (26.9%), recorded in both strata but more frequently in the canopy, highlighting its affinity for sylvatic environments and its central role in arbovirus transmission, particularly in enzootic cycles involving non-human primates. Notably, this species is a well-documented vector of YFV and MAYV [6,9,11,28,45,46,47], exhibiting ecological versatility and a dispersal capacity exceeding 11 km [48,49], which supports its role as a bridge vector between forested habitats and human-modified areas, especially in regions experiencing intense land-use change.

The detection of YFV exclusively in *Hg. janthinomys* pools reinforces its epidemiological role in the Brazilian Amazon, corroborating previous data that confirm it as the primary vector in the region [9,50,51,52,53]. Molecular analysis by RT-qPCR revealed Ct values ranging from 22.2 to 27.9 in YFV-positive pools, confirming the presence of viral RNA. It should be noted, however, that Ct values obtained from pooled samples cannot be directly interpreted as viral loads, since they may reflect the contribution of multiple infected individuals within the same pool. Metagenomic sequencing further confirmed the presence of YFV through reads assigned to the virus and partial contigs showing high protein identity (>99%) with regions of the viral polyprotein from YFV reference sequences available in GenBank. In the BLASTX analysis, the most significant hits included strains UVG67813 and AWB15005, both previously reported in Brazil. Although the number of viral reads was limited and did not allow near-complete genome assembly, these results provide robust confirmation of YFV circulation in the sampled mosquito populations.

Although no viral detection was observed in the other species investigated in this study, it is important to highlight that many of them play relevant roles in the ecology of arboviruses. The record of *Haemagogus leucocelaenus*, albeit represented by a single specimen collected in the canopy, deserves attention due to its vector competence [54] and its relevance as a primary YFV vector in the South and Southeast regions of Brazil [11,16,55,56,57,58].

Although less abundant, mosquitoes of the genus *Sabethes*, such as *Sa.* (*Sabethoides*) *chloropterus*, may contribute to YFV transmission and can act as primary vectors under specific conditions, such as during periods of reduced activity of the main vectors [9,59]. Among *Aedes* species, *Aedes* (*Stegomyia*) *albopictus* stands out as an exotic species with a wide distribution and proven vector competence for several arboviruses, including YFV [60,61,62,63,64,65,66,67]. Its high ecological plasticity allows it to function as a bridge vector between rural, forested, and urban environments [68,69,70,71]. Other species of the genus, such as *Aedes* (*Ochlerotatus*) *serratus* and *Aedes* (*Ochlerotatus*) *scapularis*, are also epidemiologically relevant and associated with transition areas, having been found naturally infected with different arboviruses, including YFV [31,72,73,74,75,76,77,78,79,80,81,82]. *Psorophora* (*Janthinosoma*) *ferox*, although aggressive and highly dispersive, showed no YFV infection in this study, consistent with recent findings [83] indicating a secondary or negligible role of this species in virus transmission, with infection confirmed primarily in *Hg. janthinomys*.

In addition to YFV, other epidemiologically relevant arboviruses were screened but not detected in the mosquito samples collected in this study. *Orthoflavivirus denguei* (DENV) is endemic in Pará, with recurrent outbreaks; between 2024 and 2025, over 15,000 probable cases were reported, including 53 in the municipality under study [84], although no active cases were recorded during the collection period, which coincided with the beginning of the rainy season, a period of higher vector density and transmission risk. SLEV and WNV have low regional incidence, with recent WNV circulation confirmed primarily in the states of Minas Gerais and Piauí, and human cases remaining sporadic and limited [85,86]; the first isolation of WNV in mosquitoes in Brazil occurred in southeastern Pará from a *Culex* (*Melanoconion*) sp. Pool [24]. CHIKV has caused significant outbreaks in Brazil since 2014; between 2024 and 2025, over 2500 probable cases were reported in Pará, including 23 in the study municipality [84], but no active cases occurred during sampling. Finally, MAYV and OROV have a history of local outbreaks, most recently in 2008 and 2018, respectively, with OROV outbreaks predominating in the region and additional cases of MAYV and CHIKV reported [28,29].

Although the absence of detection for these arboviruses was somewhat unexpected, this result should be interpreted in the context of ongoing local surveillance, conducted annually during the rainy season. This period, coinciding with increased vector densities, is historically associated with higher incidence of arbovirus cases. Continuous monitoring during this season is therefore essential for the early detection of viruses such as DENV, CHIKV, MAYV, and OROV.

It is worth highlighting the possibility of underestimating viral circulation in less abundant species or those with low infection rates, as well as the absence of molecular analysis in some species that were not tested by RT-qPCR. Additionally, seasonal and environmental factors may significantly influence both the composition of the fauna and the dynamics of viral transmission, since the investigation was conducted exclusively during the rainy season. Thus, the implementation of longitudinal studies with broader temporal and spatial coverage, including the expansion of sampling sites and the inclusion of a more representative number of species in molecular analyses, should be considered in future investigations.

In summary, the results of this study expand our understanding of vector entomofauna and arbovirus circulation in forested areas of the metropolitan region of Belém. The detection of YFV in arthropods, even in the absence of human cases or non-human primate deaths, enabled the prompt activation of surveillance efforts, including the vaccination of the local population. The confirmation of *Hg. janthinomys* as an active vector of YFV, combined with the area’s outbreak history, indicates that the region is a potential hotspot for the emergence and re-emergence of arboviruses. These findings emphasize the importance of integrated monitoring strategies that consider local ecological, historical, and entomological contexts. Maintaining systematic entomovirological surveillance actions, alongside expanding yellow fever vaccination coverage, is essential for preventing and mitigating future viral emergencies in the Amazon region, reinforcing its role as a predictive tool for early detection of both human and animal cases.

## Figures and Tables

**Table 1:** Culicidae species collected using the human-attraction method in forest strata at ground level and in the canopy.

Taxa	Ground	Canopy	Total	Frequency (%)
*Ae*. (*How.*) *fulvithorax* ♀	13	0	13	4.5
*Ae.* (*Och.*) *scapularis* ♀	3	0	3	1.0
*Ae.* (*Och.*) *serratus* ♀	26	0	26	9.1
*Ae.* (*Pro.*) *argyrothorax* ♀	6	0	6	2.1
*Ae.* (*Sal.*) *hortator* ♀	1	0	1	0.3
*Ae.* (*Stg.*) *albopictus* ♀	1	0	1	0.3
*Ae.* (*Stg.*) *albopictus* ♂	3	0	3	1.0
*Cx.* (*Car*.) sp. ♀	1	0	1	0.3
*Hg.* (*Hag.*) *janthinomys* ♀	30	47	77	26.9
*Li. durhamii* ♀	5	0	5	1.7
*Li. flavisetosus* ♀	67	0	67	23.4
*Ps.* (*Jan.*) *ferox* ♀	17	1	18	6.3
*Trichoprosopon* sp. ♀	1	0	1	0.3
*Wyeomyia* sp. ♀	54	0	54	18.9
*Hg.* (*Con.*) *leucocelaenus* ♀	0	1	1	0.3
*Sa.* (*Sbo.*) *chloropterus* ♀	0	7	7	2.4
*Sa.* (*Sbo.*) *glaucodaemon* ♀	0	2	2	0.7
Total	228	58	286	100

Legend: ♂—Male; ♀—Female.

**Table 2:** Mosquito species and number of specimens per pool, viral panel used for RT-qPCR screening, and Ct values.

Sample ID	Species (Number Per Pool)	RT-qPCR Viral Screen	Ct Value
AR888768	*Ae.* (*How.*) *fulvithorax* ♀ (13)	YFV, MAYV, OROV	-
AR888769	*Ae.* (*Och.*) *scapularis* ♀ (3)	YFV, MAYV, OROV	-
AR888770	*Ae.* (*Och.*) *serratus* ♀ (26)	YFV, MAYV, OROV	-
AR888771	*Ae.* (*Pro.*) *argyrothorax* ♀ (6)	YFV, MAYV, OROV	-
AR888772	*Ae.* (*Sal.*) *hortator* ♀ (1)	YFV, MAYV, OROV	-
AR888773	*Ae.* (*Stg.*) *albopictus* ♀ (1)	YFV, DENV, CHIKV, MAYV, OROV	-
AR888774	*Ae.* (*Stg.*) *albopictus* ♂ (3)	YFV, DENV, CHIKV, MAYV, OROV	-
AR888775	*Cx.* (*Car.*) sp. ♀ (1)	WNV, SLEV	-
AR888776	*Hg.* (*Hag.*) *janthinomys* (30) ♀ (Ground)	YFV, MAYV, OROV	YFV = 23.7
AR888777	*Li. durhamii* ♀ (5)	NA	-
AR888778	*Li. flavisetosus* ♀ (25)	NA	-
AR888779	*Li. flavisetosus* ♀ (32)	NA	-
AR888780	*Li. flavisetosus* ♀ (10)	NA	-
AR888781	*Ps.* (*Jan.*) *ferox* ♀ (17)	YFV, MAYV	-
AR888782	*Trichoprosopon* sp. ♀ (1)	NA	-
AR888783	*Wyeomyia* sp. ♀ (21)	NA	-
AR888784	*Wyeomyia* sp. ♀ (33)	NA	-
AR888785	*Hg.* (*Con.*) *leucocelaenus* ♀ (1)	YFV, MAYV, OROV	-
AR888786	*Hg.* (*Hag.*) *janthinomys* (10) ♀ (Canopy)	YFV, MAYV, OROV	YFV = 26.1
AR888787	*Hg.* (*Hag.*) *janthinomys* (27) ♀ (Canopy)	YFV, MAYV, OROV	YFV = 22.2
AR888788	*Hg.* (*Hag.*) *janthinomys* (10) ♀ (Canopy)	YFV, MAYV, OROV	YFV = 27.9
AR888789	*Ps.* (*Jan.*) *ferox* ♀ (1)	YFV, MAYV	-
AR888790	*Sa.* (*Sbo.*) *chloropterus* ♀ (7)	YFV	-
AR888791	*Sa.* (*Sbo.*) *glaucodaemon* ♀ (2)	YFV	-

Legend: ID—Sample identification; ♂—Male ♀—Female; YFV: Orthoflavivirus flavi; DENV: Orthoflavivirus denguei; WNV: Orthoflavivirus nilense; SLEV: Orthoflavivirus louisense; CHIKV: Alphavirus chikungunya; MAYV: Alphavirus mayaro; OROV: Oropouche orthobunyavirus; RT-qPCR: Reverse Transcription Quantitative Polymerase Chain Reaction; Ct: Cycle threshold; NA: Not analyzed.

**Table 3:** Results of metagenomic sequencing, taxonomic classification, and BLASTX analysis of pools positive for YFV.

						BLASTX				
Sample ID	Raw Data	Fastp	Viral Reads	YFV Reads	N° of Contigs	Region	Per. Identity (%)	QC (%)	e-Value	Genbank Accession
AR888776	37,830,530	36,550,112	50,390	54	5	pol	99.74	39	0.0	UVG67813
AR888787	40,537,094	39,359,822	37,007	12	7	pol	99.46	21	5 × 10^−105^	AWB15005
AR888788	32,571,896	31,670,072	38,178	3	NA	NA	NA	NA	NA	NA

Legend: Raw data: total number of paired raw reads generated; Fastp: number of reads retained after quality control; Viral Reads: total number of viral reads identified; YFV Reads: number of reads assigned to yellow fever virus (YFV); N°. of contigs: number of assembled contigs; Region: identified genomic region; pol: viral polyprotein; Per. identity (%): percentage identity of the protein compared to the reference sequence; QC: query coverage; e-value: statistical value of the alignment; GenBank accession: reference sequence identifier in the GenBank database; NA: not applicable, indicates absence of data.

## Data Availability

The original contributions presented in this study are included in the article/Supplementary Material. Further inquiries can be directed to the corresponding author.

## Supplementary Materials

The following supporting information can be downloaded at: https://www.mdpi.com/article/10.3390/v17091258/s1, Supplementary Material S1, RT-qPCR amplification plots illustrating viral detection; Supplementary Material S2, partial contig recovered from sample AR888776; and Supplementary Material S3, partial contig of Yellow Fever Virus (YFV) recovered from sample AR888787.

## Abbreviations

The following abbreviations are used in this manuscript:

RT-qPCR	Real-Time Reverse Transcription Polymerase Chain Reaction
YF	Yellow Fever
YFV	Yellow Fever virus
DENV	Orthoflavivirus denguei
WNV	Orthoflavivirus nilense
SLEV	Orthoflavivirus louisense
CHIKV	Alphavirus chikungunya
MAYV	Alphavirus mayaro
OROV	Oropouche orthobunyavirus
ILHV	Orthoflavivirus ilheusense
