# Supplementary figures and images for: Molecular Detection of Yellow Fever Virus in Haemagogus janthinomys Mosquitoes (Diptera: Culicidae) in a Rural Settlement in the State of Pará, Brazilian Amazon, 2024
Source: Viruses. 2025 Sep 18;17(9):1258. doi: 10.3390/v17091258 (PMC12474192; doi:10.3390/v17091258)

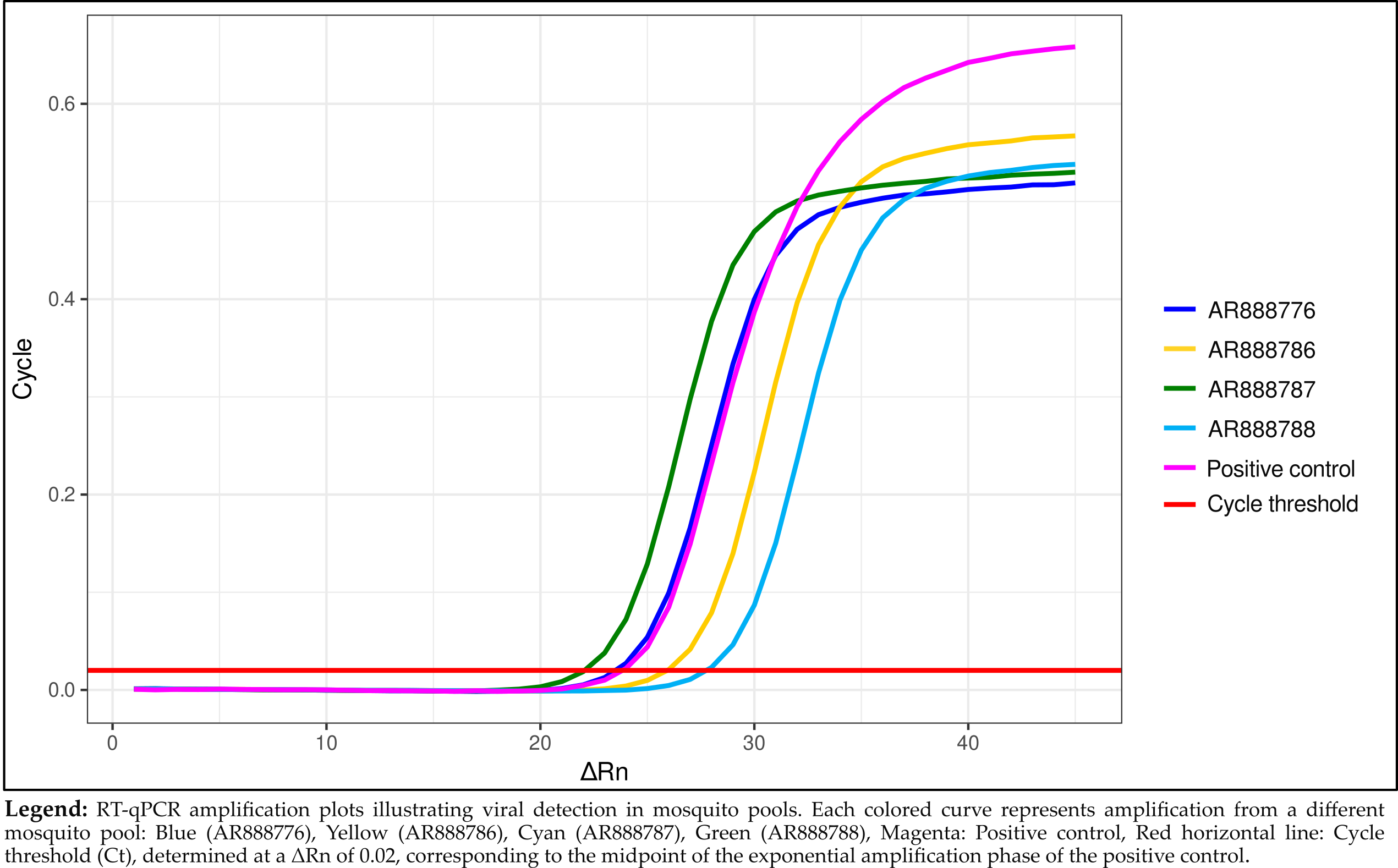

Supplement: Supplementary file 1 [file viruses-17-01258-s001.zip › Supplementary Material S1.tiff]
